# Account of Diseases in an Eastern District of London, from December 20, 1802, to January 20, 1803

**Published:** 1803-02-01

**Authors:** 


### *Account of Diseases in an Eastern District of London, from December 20, 1802, to January 20, 1803.*

| | |
|---|---|
| ACUTE DISEASES. | |
| Pneumonia - - - - | 3 |
| Peripneumonia Notha - | 5 |
| Dysenteria - - - - - | 3 |
| Febris Intermittens - - | 1 |
| Rheumatismus Acutus - | 4 |
| CHRONIC DISEASES. | |
| Catarrhus - - - - - - | 10 |
| Tussis - - - - - - - | 25 |
| Dyspnœa - - - - - - | 14 |
| Tussis cum Dyspnœa - | 12 |
| Raucedo - - - - - - | 3 |
| Asthma - - - - - - - | 1 |
| Hæmoptoe - - - - | 2 |
| Phthisis Pulmonalis - | 3 |
| Palpitatio - - - - - - | 1 |
| Hydrothorax - - - - | 3 |
| Cephalalgia - - - - | 5 |
| Vomitio - - - - - - | 2 |
| Dyspepsia - - - - - | 10 |
| Gastrodynia - - - - | 4 |
| Enterodynia - - - - - | 5 |
| Hæmorrhagia Intestinalis | 1 |
| Hæmorrhois - - - - | 2 |
| Chlorosis - - - - - | 3 |
| Amenorrhœa - - - - | 4 |
| Lumbago - - - - - - | 2 |
| Rheumatismus Chronicus | 23 |
| PUERPERAL DISEASES. | |
| Menorrhagia Lochialis - | 2 |
| Enuresis - - - - - - | 2 |
| Dolores Post Partum - | 3 |
| Ephemera - - - - - - | 2 |
| INFANTILE DISEASES. | |
| Vermes - - - - - - - | 2 |
| Febris Mesenterica - - | 1 |
| Tinea - - - - - - - - | 2 |

The state of diseases, as it may well be supposed, has varied during the last few weeks in consequence of the great changes which have taken place in the weather. The temperature of the air has been very different and the changes have been rapid. After a series of very mild weather the frost appeared very suddenly, and gave occasion to many of those complaints which usually occur at this season of the year. Diseases of the chest are daily increasing in number; Coughs, Catarrhs, and Peripneumonic affections are becoming very general; and many aged persons, who had passed through a portion of the winter with tolerable comfort, are now suffering in consequence of the severity of the weather.

The Bill of Mortality states the number of deaths by the Small-pox during the last year as larger than that in the preceding. We were happy, however, to observe, that in that year the number was less than in former ones by nearly 1000. The comparison, therefore, of the present with former lists, affords a pleasing prospect of the diminished effects of this dreadful disease. It is of still more importance however, to remark that the subject has lately excited much of the public attention, and has produced such vigorous exertions in order to lessen the influence, or to procure the entire extermination of the disease, that it may be hoped it will soon cease to find a place in the list of those maladies by which the human frame has been visited.

